# Dickkopf-3: Current Knowledge in Kidney Diseases

**DOI:** 10.3389/fphys.2020.533344

**Published:** 2020-12-16

**Authors:** Xiangdong Fang, Jing Hu, Yanxia Chen, Wen Shen, Ben Ke

**Affiliations:** ^1^Department of Nephrology, The Second Affiliated Hospital of Nanchang University, Nanchang, China; ^2^The Third Hospital of Nanchang, Nanchang, China; ^3^Department of Cardiovascular Medicine, The Second Affiliated Hospital of Nanchang University, Nanchang, China

**Keywords:** Dickkopf-related protein 3, wnt signaling pathway, chronic kidney disease, idiopathic membranous nephropathy, biomarker

## Abstract

Dickkopf-related protein 3 (DKK3) is a secreted glycoprotein that has been implicated in the pathogenesis of a variety of diseases. Recent evidence suggests that urinary DKK3 may serve as a potential biomarker for monitoring kidney disease progression and assessing the effects of interventions. We review the biological role of DKK3 as an agonist in chronic kidney disease (CKD) and autosomal dominant polycystic kidney disease (ADPKD) and as an antagonist in idiopathic membranous nephropathy (IMN). In addition, we present the clinical applications of DKK3 in acute kidney disease and tubulointerstitial fibrosis, suggesting that urine DKK3 may be a potential biomarker for acute kidney disease and CKD. Further research into the mechanism of DKK3 and its use as a diagnostic tool, alone or in combination with other biomarkers, could prove clinically useful for better understanding the pathology of kidney diseases and improving early detection and treatment.

## Introduction

Dickkopf (DKK) proteins constitute an evolutionarily conserved family that includes five secreted glycoproteins: DKK1–4, which share two conserved cysteine-rich domains (CRDs), and a divergent member, soggy (Glinka et al., 1998). The DKK family is characterized by the N-terminal CRD, which is not found in other vertebrate proteins. The two CRDs are separated by a linker region, which is similar in DKK1, -2, and -4 but is significantly shorter in DKK3 (Krupnik et al., 1999). Since their discovery, several studies have implicated DKK proteins in human disease, principally cardiovascular diseases (Baetta and Banfi, 2019), cancer (Shen et al., 2020), arthritis (Mu et al., 2020), macular degeneration (Tom et al., 2020), and Alzheimer’s disease (Shi et al., 2020). DKK proteins are co-ordinately found in mesenchymal lineages in embryogenesis (Monaghan et al., 1999), which also leads to kidney development (Stark et al., 1994).

Dickkopf-related protein 3 (DKK-3) is a secreted glycoprotein with a molecular weight of 38 kDa that is synthesized by stressed tubular epithelia and is significantly expressed in mesenchymal progenitor and mesenchymal cells *in vitro* (Meister et al., 2015; Federico et al., 2016). DKK3 is a multifunctional protein involved in various cellular processes, such as cell differentiation, proliferation and apoptosis, via the Wnt/β-catenin pathway (Veeck and Dahl, 2012) and contributes to multiple diseases, including cancer (Liu et al., 2020b), chronic heart failure (Cao et al., 2018), and acute myeloid leukemia (Xu et al., 2017). The Wnt/β-catenin pathway is one of the important signaling pathways leading to kidney disease (Ke et al., 2017). Wnt molecules, in the canonical Wnt/β-catenin signaling pathway, interact with Frizzled receptors and the co-receptor LDL receptor-related protein (Lrp) 5/6 to transmit the intracellular signal in the intracellular matrix. Then, the interaction activates an intracellular signaling cascade contributing to an accumulation of non-phosphorylated β-catenin, which translocates into the cell nucleus, triggering the transcription of Wnt target genes by working with the transcription factors T cell factor (TCF)/lymphoid enhancer-binding factor (LEF) (Miao et al., 2019). Thus, the persistent accumulation of intracellular β-catenin activates the Wnt/β-catenin signaling pathway, which has been implicated in the development of renal fibrosis (Miao et al., 2019), nephropathy-related osteoporosis (Liu et al., 2020a), podocyte injury and proteinuria, persistent tissue damage during acute kidney injury and cystic kidney diseases (Schunk et al., 2020). It has been demonstrated that DKK1, -2, and -4 directly interact with the Wnt/β-catenin pathway by binding Lrp-5/6 coreceptors (van Andel et al., 2019), while DKK3 neither binds Lrp nor Krm coreceptors, a ternary complex that leads to internalization and degradation of Lrp, at the cell surface membrane (Veeck and Dahl, 2012). Therefore, DKK3 has not precisely been associated with Wnt signaling (Hamzehzadeh et al., 2018). In addition to Wnt signaling, DKK3 contributes to renal injury in multiple ways (Lipphardt et al., 2019). DKK3, whether in urine or tissue, may be a potential biomarker to monitor kidney disease progression and assess the effects of interventions (Zewinger et al., 2018; Schunk et al., 2019). Here, we summarize recent novel findings regarding the implications for DKK3 in kidney diseases.

## DKK3 and Chronic Kidney Disease

Chronic kidney disease (CKD) is defined as decreased kidney function shown by a GFR of less than 60 mL/min per 1⋅73 m^2^, markers of kidney damage, or both, lasting at least 3 months, regardless of the underlying cause. The final and common pathological consequence of CKD is renal fibrosis, which is characterized by fibroblast proliferation, endothelial–mesenchymal transition (EMT) and the accumulation of extracellular matrix (ECM) (Ke et al., 2015).

**FIGURE 1:**
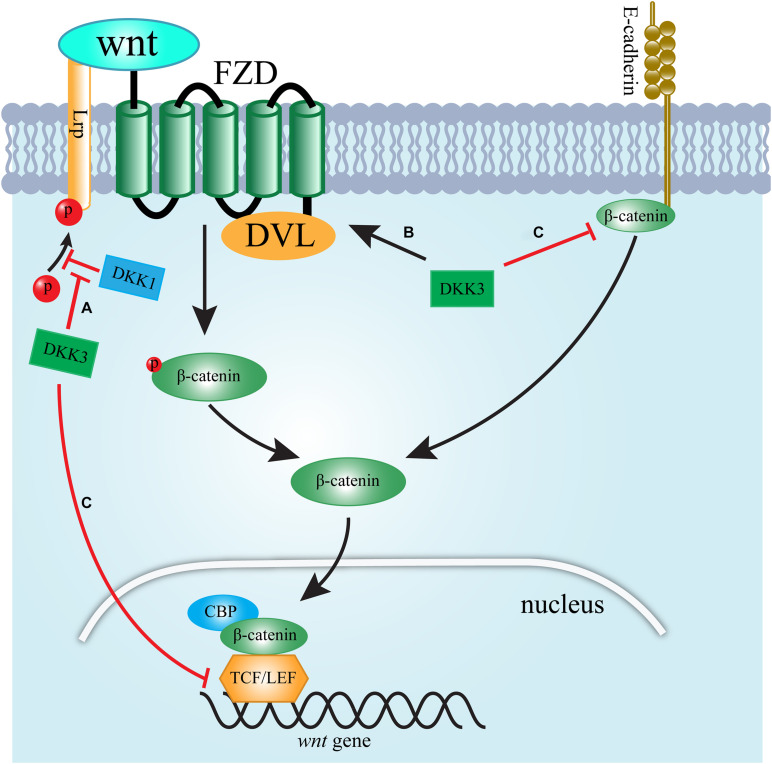
Schematic presentation of the signaling pathways by which DKK3 regulates WNT signaling. **(A)** DKK3 supports the activation of Wnt signaling by counteracting the antagonistic effects of DKK1 binding to LRP5/6. **(B)** DKK3 activates Wnt signaling by increasing the activation of the FZD/DVL interaction. **(C)** DKK3 inhibits Wnt signaling via downregulating membrane-bound β-catenin expression and TCF/LEF activity.

Dickkopf-related protein 3 accelerates renal fibrosis through the Wnt signaling pathway (Figure 1). The ability of DKK3 to modulate Wnt signaling is controversial and depends on the tissue context (Federico et al., 2016). Studies have revealed that DKK3 promotes renal fibrosis by supporting the activation of Wnt signaling (Federico et al., 2016; Lipphardt et al., 2019). DKK1 is known as an inhibitor of canonical Wnt/β-catenin signaling (Lipphardt et al., 2019). Lipphardt, Dihazi (Lipphardt et al., 2019) found that DKK3 activates the Wnt pathway by counteracting the antagonistic effects of DKK1 binding to LRP5/6. Intriguingly, fibroblast sensitivity to DKK3 increased significantly at lower concentrations of DKK1, suggesting that at locations far away from the dysfunctional endothelial cells secreting DKK3, DKK1 plays a dominant role, and Wnt signal is partially counteracted^11^. Endothelial-secreted DKK3 has more influence than DKK1 at the sites nearest to dysfunctional endothelial cells, such as pericytes (Lipphardt et al., 2019). Thus, interstitial fibroblasts may be more affected by DKK3 secreted by the epithelium, which is able to convert fibroblasts to myofibroblasts and result in renal fibrosis (Lipphardt et al., 2019). Moreover, Federico et al. (2016) also found that DKK3 mediates tubular atrophy and interstitial fibrosis by activating the Wnt pathway. They found that the low expression of DKK3 improves renal tubular atrophy, and reduces the accumulation of interstitial matrix in two mouse models of renal fibrosis by decreasing activation of Frizzled receptors (Federico et al., 2016). However, there is a contrary view. β-catenin not only acts as a transcription factor but is also one of the essential scaffold proteins that stabilize tubular epithelial architecture via binding E-cadherin. It is suggested that β-catenin dissociates from the cell membrane and gives rise to the cytosolic-free β-catenin equilibrium pool. Once stabilized, cytosolic β-catenin translocates to the cell nucleus and promotes target gene transcription (Wong et al., 2016). A study reported that in overload albuminuria mice, β-catenin shows a dual character in renal. In this study, the expression of β-catenin was upregulated at the early stage after disease induction (4 weeks) but was terminated at the late stage (8 weeks), which was accompanied by significant tubular apoptosis (Wong et al., 2016). Notably, there was an nuanced relationship between DKK-3 and renal tubular β-catenin expression at these period (Wong et al., 2016). The increased expression of DKK-3 was observed after longtime exposure to albumin, contributing to inhibition of the intracellular β-catenin signaling pathway (Wong et al., 2016). This study confirmed the relationship between DKK-3 and β-catenin signaling in HK-2 cells by incubation with exogenous DKK-3 (Wong et al., 2016). These results suggest that DKK3 induces tubular cell apoptosis in proteinuric nephropathy by downregulating membrane-bound β-catenin expression and the activity of the transcription factor T-cell factor (TCF)/lymphoid enhancer-binding factor (LEF) promotor.

Dickkopf-related protein 3contributes to chronic renal injury through other pathways. Vascular damage is one of essential factors contributing to renal fibrosis (Ke et al., 2015). A study found that in renal microvascular endothelial cells, DKK3 significantly reduces the total length, area percentage and bifurcation number of capillary-like structures (Lipphardt et al., 2019). Moreover, adriamycin-induced nephropathy resulted in the up-regulation of DKK3 expression in the tubular, but not in endothelial compartments. Thus, DKK3 induces EMT and impairs angiogenic competence to give rise to renal fibrosis (Lipphardt et al., 2019). In addition, DKK3 stimulated the expression of transforming growth factor-β (TGF-β) (Karamariti et al., 2018), which is recognized as one of the most important factors leading to renal fibrosis. DKK3 influences chronic inflammatory fibrotic kidney disease by facilitating the inflammatory T cell response (Federico et al., 2016). The Th2 response in wild-type mice with UUO switched to Th1 T cell activity accompanied by an increase in cytokines, such as IL-1β, IFNγ, and TNFα. Elevated expression of INFγ is associated with inhibition of increased matrix production; one potential mechanism is the repressive action of INFγ on the transcription of collagen genes (Grone et al., 2017). A study showed that DKK3 modulates the local T cell response in renal fibrosis by changing the polarization of T cell, as shown via an increase in IFNγ-producing T cells (Federico et al., 2016).

Taken together, despite the relationship between DKK3 and WNT signaling, DKK3 results in a fibrogenic lesion in the kidney. Moreover, DKK3 facilitates renal fibrosis by promoting EMT, impairing angiogenic competence, activating TGF-β, and modulating local T cell responses.

## DKK3 and Acute Kidney Injury

Acute kidney injury (AKI) is defined as an increase in serum creatinine (SCr) of at least 0.3 mg/dL within 48 h; an increase of more than 1.5 times the baseline level of SCr, which is known or presumed to have occurred within the prior 7 days; or a decrease in 6-h urinary output (UO) below 0.5 ml/kg/h. AKI is a common complication in hospitalized patients and is associated with poor short-term and long-term prognosis (Chawla et al., 2017). Considering the impact of AKI on short- and long-term outcomes, it is of high importance to understand the mechanism of AKI. The Wnt/β-catenin pathway is transiently activated and recognized as a protective response, which can minimize cell damage by promoting tubular repair and regeneration after AKI (Zhou et al., 2017). In addition, Wnt agonists improved renal regeneration and function after kidney ischemia-reperfusion (I/R), and reduced inflammation and oxidative stress (Kuncewitch et al., 2015). Thus, activation of Wnt/β-catenin signaling may serve as a therapeutic target in AKI.

Dickkopf-related protein 3 induces AKI by inhibiting Wnt/β-catenin signaling. Zhu et al. (2018) found that the expression of β-catenin is significantly increased after inhibiting *dkk3* expression in I/R-induced cell and rat AKI models. Therefore, the expression of DKK3 is positively correlated with AKI. However, it should be mentioned that the exaggerated and continuous activation of Wnt/β-catenin pathway may contribute to the transition of AKI to chronic kidney disease (CKD) (Xiao et al., 2016).

## DKK3 and Autosomal Dominant Polycystic Kidney Disease

Autosomal dominant polycystic kidney disease (ADPKD) is the most common monogenic kidney disease worldwide, affecting one in 500–1,000 births (Messchendorp et al., 2019). The focal development of renal cysts in an age-dependent manner is unique to ADPKD. Generally, in the first three decades of ADPKD, only a few renal cysts are clinically detectable; however, thousands of renal cysts of different sizes can be found in most ADPKD by the fifth decade (Messchendorp et al., 2019). Progressive cyst expansion with age leads to massive enlargement and distortion of the normal architecture of both kidneys and, ultimately, ESRD in most patients. ADPKD is also associated with a high risk for cardiac valvular diseases and glucose and lipid metabolism abnormalities (Fliszkiewicz et al., 2019; Messchendorp et al., 2019). However, the mechanism that modulates renal disease progression in ADPKD is poorly understood.

Genetic variations in DKK3 may be involved in ADPKD. Liu et al. (2010) found that three single-nucleotide polymorphisms at the *dkk3* gene locus showed a significant association with eGFR in ADPKD. The SNP rs3750940 shows the strongest association with eGFR in ADPKD (accounting for 1.4% of the total variance) (Liu et al., 2010). Wnt signaling, which has been shown to cause PKD, is downregulated by DKK3 and may account for this (Qian et al., 2005; Liu et al., 2010).

## DKK3 and Idiopathic Membranous Nephropathy

In recent years, idiopathic membranous nephropathy (IMN) has attracted extensive attention due to the development of autoantibodies, anti-phospholipase A2 receptor and anti-thrombospondin type I domain-containing 7A on podocytes, the establishment of circulating immune network complexes and the development of autoreactive immune cells against the kidney, both innate and adaptive (van de Logt et al., 2019). The autoinflammatory responses in IMN lead to dysfunction of glomerular cells. Therefore, immune system-mediated mechanisms play an essential role in idiopathic membranous nephropathy (Motavalli et al., 2019).

**FIGURE 2:**
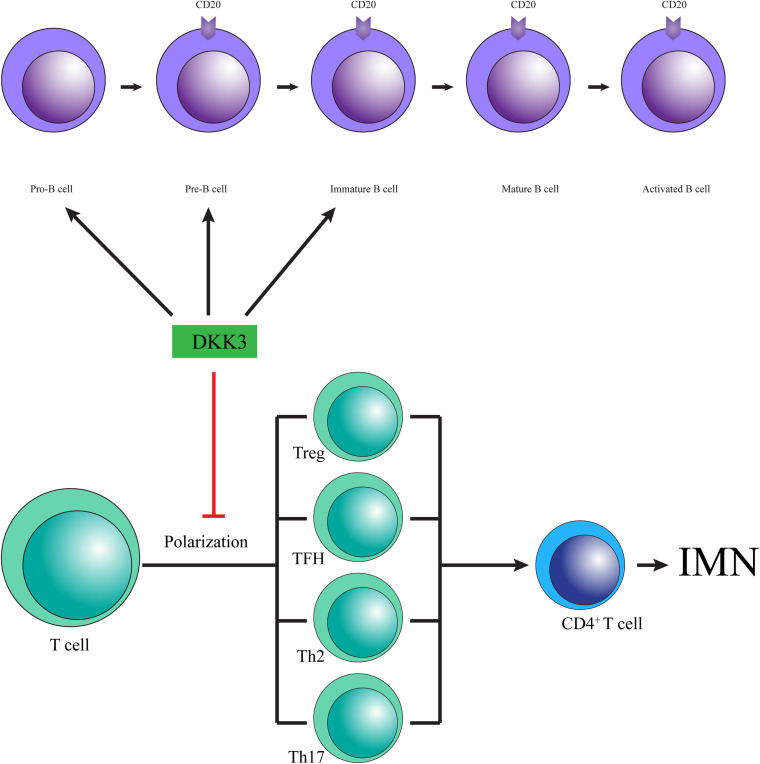
The possible mechanisms by which DKK3 is involved in IMN.

Dickkopf-related protein 3 may prevent IMN by changing T cell polarization (Figure 2). Since discovered, the anti-PLA2R and anti-THSD7A autoantibodies promote a paradigm shift in IMN from a histological to pathophysiological pattern and change our empirical treatment by preventing antibody production (Ronco and Debiec, 2019). T cells, a crucial factor in the immune response, conduct immune homeostasis and tolerance by supporting the release of cytokines, such as IFNγ and IL-10, the promotion of B cells, and the recruitment of macrophages, neutrophils, natural killer (NK) cells and other subgroups of T helper cells. A lack of tolerance facilitates autoantibody development and induces inflammation. Ultimately, T cell-derived cytotoxicity results in tissue damage, particularly in the kidney in IMN (Motavalli et al., 2019). Meister et al. (2015) showed that low expression of DKK3 promoted the aggravation of experimental autoimmune response, in which T cell polarization induced by an increase in IFNγ producing T cells was observed in the central nervous system. Moreover, DKK3 deficiency increased numbers of CD3-positive T cells in kidneys, accompanied by increased levels of IFNγ- and TNFα-producing CD4 + and CD8 + T cells (Federico et al., 2016), and exogenous DKK3 reduced CD8 T cell reactivity (Papatriantafyllou et al., 2012). These results suggest that DKK3 may relieve IMN by affecting T cell polarization and decreasing the expression of IFNγ.

Dickkopf-related protein 3 may attenuate IMN by modulating the fate and function of B cells (Figure 2). The imbalance of B cells in the immune response is considered to be a main cause of autoimmune disease, which leads to the neglected presentation of autoantigens, the production of autoantibodies and the impairment of cytokine secretion (Motavalli et al., 2019). Moreover, a high percentage of B lymphocytes has been reported in IMN [22]. Recent reports revealed that inhibition of B cell proliferation and development of pathogenic antibody effectively alleviated the progress of IMN (Fervenza et al., 2019). Thus, the stability and development of B cells acts as an essential role in the pathogenesis of IMN. Julia Ludwig and colleagues found that the composition of the B cell compartment is changed in DKK3-deficient mice, in which DKK3 is capable of altering the development and maintenance of B cells. The development of B2 cells was repressed in adult DKK3-deficient mice at the pre- and immature B cell stages, contributing to decreased numbers of follicular B cells, which represent the majority of B cells in the body (Ludwig et al., 2015). Furthermore, DKK3 destroyed the self-maintenance of peripheral B1 cells by reducing the survival and proliferation of B1 cells (Ludwig et al., 2015). In addition, DKK3-deficient mice showed significantly increased expression of the cytokine IL-10, which is surprisingly increased in IMN (Kuroki et al., 2005; Ludwig et al., 2015). Therefore, DKK3 is a vital regulatory protein for the development of a normal B cell compartment.

Collectively, DKK3 may be a potential therapeutic target in the treatment of IMN because it changes T cell polarization, maintains B cell development and function, and decreases the cytokines released by T cells and B cells. However, there have been few studies on the role of DKK3 in IMN.

## Clinical Application of DKK3 in Kidney Diseases

The presence of DKK3 may serve as a biomarker of kidney diseases. In kidney tissue, DKK3 promotes the progression of AKI and CKD. In urine, DKK3 also displays a potential biomarker for AKI and CKD (Zewinger et al., 2018; Schunk et al., 2019). For example, in patients undergoing cardiac surgery, concentrations of DKK3 in urine relative to creatinine that were higher than 471 pg/mg were independently associated with a increased risk of AKI (Schunk et al., 2019). Moreover, urinary concentrations of DKK3 relative to creatinine significantly improved AKI prediction compared with clinical and other laboratory measurements (Schunk et al., 2019). Thus, Schunk et al. (2019) concluded that high level of urinary DKK3 was associated with great risk of AKI, preoperative urinary DKK3 not only predicted the risk of AKI and decrease of eGFR at hospital discharge, elevated baseline urinary DKK3 was also associated with a significant decrease in eGFR and an increased risk of a significant decrease in eGFR during long-term follow-up in the study, and urinary DKK3 might be a novel tool to identify patients who might benefit from specific preventive strategies. Another study showed that in patients with CKD, median urinary DKK3-to-creatinine concentration at baseline was prominently higher than in the general population (Zewinger et al., 2018). Urinary DKK3 dramatically improved the prediction of kidney function decline compared with that of eGFR or albuminuria alone (Zewinger et al., 2018). Urinary DKK3-to-creatinine levels were closely related to the extent of tubulointerstitial fibrosis in kidney biopsies (Zewinger et al., 2018). Hence, urinary DKK3 levels show a high sensitivity in patients who are at great risk for eGFR decline despite the cause of kidney injury, and DKK3 is a potential tool to monitor CKD progression and assess the effects of interventions (Zewinger et al., 2018). Taken together, DKK3, whether in urine or tissue, is a potential biomarker to monitor kidney disease progression and assess the effects of interventions.

## Conclusion

Dickkopf-related protein 3 has a negative role in CKD, although the ability of DKK3 to modulate Wnt signaling is controversial. Moreover, genetic variation in DKK3 modifies the severity of ADPKD, in which Wnt signaling is involved. However, DKK3 may play a renoprotective role in IMN by changing T cell polarization, maintaining B cell development and function, and decreasing the cytokines released by T cells and B cells, which needs to be confirmed in basic and clinical trials. Notably, DKK3 has shown powerful clinical value and should be used in the clinic to predict the diagnosis of kidney diseases; however, multicentre trials are still needed. On the one hand, urinary concentrations of DKK3 relative to creatinine were associated with a significantly increased risk for AKI and improved AKI prediction. On the other hand, urinary DKK3-to-creatinine levels were related to the extent of tubulointerstitial fibrosis and significantly improved the prediction of kidney function decline compared with that of eGFR or albuminuria alone. Taken together, translational studies that assess and modulate DKK3 may lead to new avenues for the prognosis, prevention and treatment of kidney diseases.

## Author Contributions

WS and BK: substantial contributions to the conception of the work, revising the article critically for important intellectual content, final approval of the submitted version, and both agreed to be accountable for all aspects of the work in ensuring that questions related to the accuracy or integrity of any part of the work are appropriately investigated and resolved. XF: drafting the article, final approval of the version to be published, and agrees to be accountable for all aspects of the work in ensuring that questions related to the accuracy or integrity of any part of the work are appropriately investigated and resolved. JH and YC: searching the literature and collecting related information. All authors read and approved the final manuscript.

## Conflict of Interest

The authors declare that the research was conducted in the absence of any commercial or financial relationships that could be construed as a potential conflict of interest.
